# Wide Dissection Trans-Sulcal Approach for Resection of Deep Intra-Axial Lesions in Eloquent Brain Areas

**DOI:** 10.3390/curroncol29100581

**Published:** 2022-10-04

**Authors:** Brandon Kaye, Raphael Augusto Correa Bastianon Santiago, Gerard MacKinnon, Rocco Dabecco, Bilal Ibrahim, Assad Ali, Romel Santos, Phillip Johansen, Surabhi Ranjan, Michal Obrzut, Hamid Borghei-Razavi, Badih Adada

**Affiliations:** 1 Dr. Kiran C. Patel College of Allopathic Medicine, Nova Southeastern University, Davie, FL 33328, USA; 2 Department of Neurosurgery, Cleveland Clinic Florida, Weston, FL 33331, USA

**Keywords:** trans-sulcal approach, surgical outcome, eloquence, glioblastoma, language, brain metastasis, surgical technique

## Abstract

*Introduction:* Resection of intra-axial tumors (IaT) in eloquent brain regions risks major postoperative neurological deficits. Awake craniotomy is often used to navigate these areas; however, some patients are ineligible for awake procedures. The trans-sulcal approach (TScal) was introduced to reduce parenchymal trauma during tumor resection. We report our experiences utilizing TScal for resection of deep IaT located in eloquent areas. *Materials and Methods:* This is a single-center retrospective analysis of patients who underwent IaT resection in eloquent areas via TScal from January 2013 to April 2021. Seventeen cases were reviewed, and relevant data was collected. Fluorescence-guided surgery with 5-aminolevulinic acid (ALA) and intraoperative ultrasound was performed in some cases. *Results:* Seventeen patients (10 males, 7 females) averaging 61.2 years-old (range, 21–76) were included in this study. Average length of stay was 4.8 days, and only 2 patients (11.8%) required hospital readmission within 30 days. Gross total resection (GTR) was achieved in 15 patients (88.2%), while subtotal resection occurred in 2 patients (11.8%). Eleven patients (64.7%) reported full resolution of symptoms, 4 patients (23.5%) reported deficit improvement, and 2 patients (11.8%) experienced no change from their preoperative deficits. No patient developed new permanent deficits postoperatively. Discussion: GTR, preoperative deficit reduction, and complications were comparable to awake craniotomy and other TScal studies. Ancillary intraoperative techniques, such as brain mapping, 5-ALA and intraoperative ultrasound, are afforded by TScal to improve resection rates and overall outcomes. *Conclusions:* TScal can be an option for patients with deep lesions in eloquent areas who are not candidates for awake surgeries.

## 1. Introduction

The resection of subcortical intra-axial tumors (IaT) in eloquent brain areas presents significant challenges, including the risk of postoperative neurological deficits [1,2,3,4,5,6]. Resecting these lesions involves traversing critical white matter tracts, and conventional transcortical resection can present technical challenges while attempting to conserve eloquent brain. Furthermore, as extent of resection (EOR) for lesions positively correlates with improved patient outcomes, a variety of techniques are necessary to increase access to lesions hidden within eloquent tissue [7,8,9,10,11,12,13]. Multiple methods have been developed to meet this need, including awake craniotomies, intraoperative neuromonitoring, and tubular retractors for both trans-cortical and trans-sulcal approaches [14,15,16,17,18,19,20]. Despite these advancements, the resection of eloquent region lesions remains an arduous task with many risks to the patient.

Awake craniotomy with neurophysiologic mapping was a major development in tumor resection in eloquent tissue, significantly decreasing postoperative deficits [21,22,23]. However, this technique is time-consuming, subject to error, and, despite adequate mapping, some lesions remain difficult to reach safely [24]. Negative neurophysiologic mapping upon stimulation of tissue does not guarantee patients will be without deficit following further resection of tissue [25,26]. In addition, adequate patient communication is necessary to perform this technique. Error is introduced with pediatric patients and those that are unable to follow commands [27,28,29]. Intraoperative seizures are a common concern when applying electrical stimulus to the brain, further complicating patient communication [30]. Awake craniotomy has also been associated with anxiety and PTSD-related symptoms following surgery, although these observations are still being investigated [31,32,33]. Awake craniotomy is a revolutionary technique but is not indicated for all patients.

The trans-sulcal approach (TScal) intends to offer an alternative method to preserving eloquent cortex, without the need for awake mapping. Theoretically, this approach preserves the projection fibers of the cortex at the expense of short associating fibers [1,34]. Projection fibers are white matter fibers that are perpendicular to the cortical surface. The fibers often traverse across hemispheres, as well as within tracts to and from the peripheral nervous system and deeper brain structures. Short association fibers, also referred to as “U-fibers” or arcuate fibers, are short white matter fibers that lie at the floor of sulci and serve as cortical connections between neighboring gyri [35,36,37,38]. These fibers are either cortical or directly subcortical and make up the majority of white matter connections of the brain [35]. Although short association fibers cannot be mapped by traditional DTI sequences, recent work by Catani, et al. and Attar, et al. utilizing ultra-high resolution DTI has mapped conserved tracts in the frontal, parietal, and occipital lobes [36,39,40]. In the trans-sulcal approach, these short fibers are crossed to preserve the gyral cortex. With improvements in mapping techniques for short association fibers, their physiological roles and association with pathological states are still emerging [41,42,43,44].

In this single-center retrospective analysis, we report two surgeons’ experiences utilizing an approach through the trans-sulcal corridor with wide dissection for the resection of IaT located in eloquent regions. This approach is technically straightforward, does not require the use of intraoperative monitoring or awake craniotomy, and avoids dissection through important white matter tracts. We aim to demonstrate the efficacy of this approach through retrospective analysis of patient outcomes and two illustrative clinical cases.

## 2. Methods

### 2.1. Patient Data

In this study, the authors retrospectively reviewed all patients who underwent resection of IaT between 1 January 2013 and 30 April 2021. Inclusion criteria for the study included patients who underwent TScal tumor resection, intra-axial and supratentorial lesion location, location of the tumor in an eloquent region, and identification of the lesion as a primary CNS tumor or distant metastasis. Eloquent regions were defined as the bilateral precentral gyri, bilateral postcentral gyri, left frontal operculum, left superior temporal gyrus, and bilateral visual cortices. Exclusion criteria included extra-axial or infratentorial lesion location and non-tumor lesion type. One-hundred and twenty-six cases were reviewed, and 17 cases met the inclusion criteria. The medical records were reviewed and deidentified data was collected on patient demographics, lesion location, lesion size, preoperative symptoms, operative details, gross total versus subtotal resection, postoperative complications, new or residual neurologic deficits, adjuvant therapy, tumor recurrence, and survival. Gross total resection (GTR) was classified as removing all of the lesion as is measured with imaging and intraoperative tumor localization. Subtotal resection was classified as resecting a lesion as much as possible while minimizing iatrogenic effects, potentially at the expense of complete excision.

### 2.2. Procedure

Preoperative assessment of all patients included contrast-enhanced MRI and CT for lesion evaluation, approach planning, target sulcus selection, neuronavigational mapping, and ultrasound. Additionally, DTI tractography and fMRI were used when deemed necessary to map important white matter tracts and assure the TScal would not cross them. Awake craniotomy was not performed in any case, and all procedures were performed under general anesthesia without intraoperative neuromonitoring. All patients received administration of mannitol, levetiracetam, and dexamethasone as per this institution’s standard procedures.

**Figure 1:**
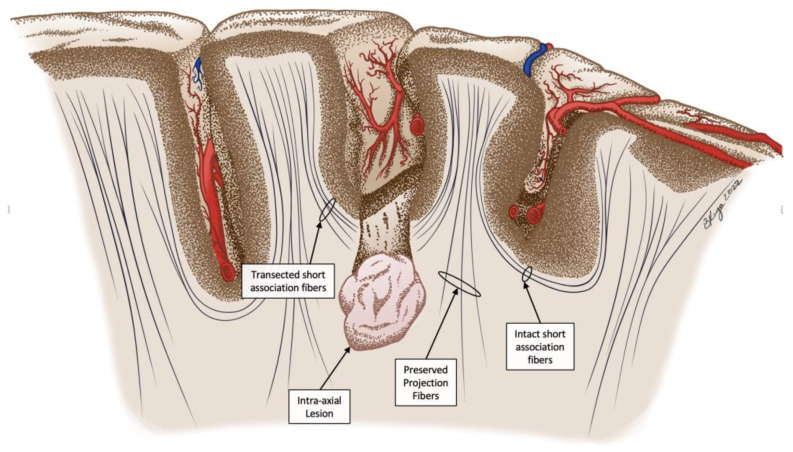
Illustration demonstrating an overview of the trans-sulcal approach employed for these subjects. A small cortectomy at the floor of the sulcus with transgression of the underlying short association fibers to reach the intra-axial lesion is shown. Preservation of gyral cortex and the projection fibers that coarse alongside the lesion is intended to preserve neurological function.

Neuro-navigation was used to outline the craniotomy overlying the target sulcus, which was performed in the typical fashion. The dura was opened, and the target sulcus was identified utilizing both anatomical landmarks and neuro-navigation. Intraoperative ultrasound (IOUS) was used supplementally to ensure proper craniotomy placement and sulcus identification before advancing further. No other intraoperative imaging modalities were utilized during the procedures. Careful microsurgical dissection of the overlying arachnoid was followed by blunt dissection of the sulcal artery and veins, which were then padded with cottonoids for protection. Further blunt dissection was performed utilizing the larger window to one side of the sulcal vessels until the floor of the sulcus was reached. At the floor of the sulcus, location over the lesions was confirmed using neuro-navigation, after which a small corticectomy of the short association fibers at the sulcal nadir was performed and the lesion was visualized (Figure 1).

**Figure 2:**
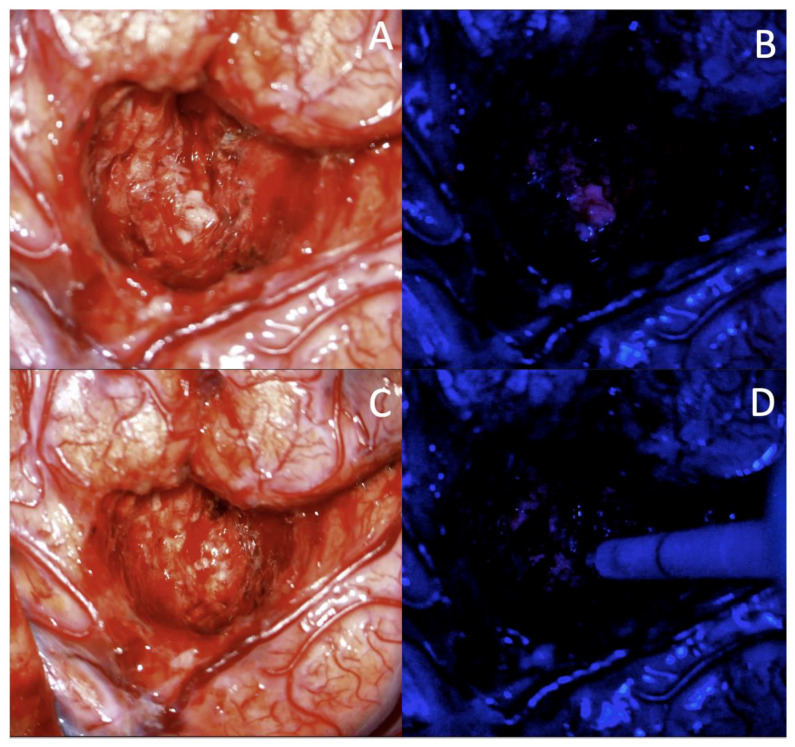
(**A**) Intraoperative visualization of High-grade glioma through a sulcus. (**B**) with fluorescent residual tumor located using 5-ALA. (**C**) Without 5-ALA fluoroscopy, achieving total resection would be impaired due to difficulty differentiating malignant from non-neoplastic tissue. After identifying the residual lesion and removing the remaining tissue, the residual cavity through the sulcus is seen and (**D**) the reduced fluorescence indicates removal of all visible malignant tissue.

Once adequate visualization of the lesion was achieved, tumor debulking and resection was performed utilizing both bipolar cautery and suction or a Cavitron ultrasonic surgical aspirator. Infiltrative lesions such as gliomas were excised using supramarginal resection and well-circumscribed metastases were removed en-bloc. For some glioma surgeries, 5-aminolevulinic (ALA) fluoroscopic guidance with a microscope filter was utilized as an adjunct for the visualization of remaining malignant tissue (Figure 2). No self-retaining retractors were utilized during any procedure; only dynamic retraction with surgical instruments and cottonoids was used on the sulcal walls. Patients were all transferred to the neuro-ICU for recovery after surgery.

## 3. Results

### 3.1. Patient Demographics

After screening for inclusion criteria, 17 patients were included in this study including 10 males and 7 females. Patient ages ranged from 21 to 76 years old, with a mean age of 61.2. Individual patient characteristics are included below (Table 1).

Only lesions located in eloquent regions as defined above were included. Lesions documented in this study were localized in the left precentral gyrus (*n* = 3), right precentral gyrus (*n* = 4), left postcentral gyrus (*n* = 1), left frontal speech area (*n* = 2), left posterior speech area (*n* = 5), and left visual cortex (*n* = 1). Both primary CNS tumors and distant metastasis were included, including glioblastoma multiforme (*n* = 12), diffuse astrocytoma (*n* = 1), small cell lung cancer (*n* = 1), lung adenocarcinoma (*n* = 2), and colorectal adenocarcinoma (*n* = 1). Of the 13 patients with gliomas, 11 presented with WHO grade 4 GBM (84.6%), 1 with a grade 3 astrocytoma (7.7%), and 1 grade 2 diffuse astrocytoma (7.7%). Two out of thirteen patients with primary CNS tumors had a prior medical history of cancer (15.4%), while all 4 patients with distant metastasis were previously diagnosed with cancer (Table 2).

Lesions were located at a depth ranging from 2 to 20 mm from the cortical surface with a median depth of 6 mm. Lesion size ranged from 15 to 64 mm, with an median diameter of 26.5 mm. Two patients were taken for unplanned (urgent or emergent) procedures (11.8%), while the remaining 15 patients underwent scheduled resection (88.2%).

Neurologic symptoms were categorized by cognitive, speech, motor, and sensory deficits. Preoperatively, 4 patients had cognitive deficits (23.5%), 12 patients had speech deficits (70.6%), 9 patients had motor deficits (52.9%), and 5 patients had sensory deficits (29.4%).

### 3.2. Operative Results

GTR was achieved in 15 patients (88.2%), while subtotal resection occurred in 2 patients (11.8%). Average length of hospital stay was 4.8 days, and 2 patients (11.8%) required hospital readmission within 30 days.

Over a 30 day-postoperative period, 15 (88.2%) patients experienced some improvement in their preoperative neurologic symptoms, and 11 (64.7%) patients had full resolution of their symptoms. Two (11.8%) patients experienced no improvements in their preoperative deficits. Three of the 4 patients with preoperative cognitive deficits experienced resolution of their symptoms. Twelve patients had preoperative speech deficits, with 11 of these patients experiencing symptom resolution. Of the 9 patients with preoperative motor symptoms, 6 patients had resolution of their symptoms, 1 patient had improvement without complete resolution, and 2 patients had persistent motor symptoms. Four of the 5 patients with preoperative sensory deficits experienced resolution of their symptoms, while 1 patient had persistent sensory deficits. New-onset transient deficits developed postoperatively in 3 patients (17.6%), however all resolved without intervention (Table 3). No patient developed new permanent deficits postoperatively.

Immediate postoperative complications included 1 infection, 2 subdural hematomas, and 3 deep vein thromboses. Additionally, 6 patients (35.3%) developed new-onset seizures within 30 days of surgery despite prophylactic levetiracetam administration. These patients’ seizures were well-controlled with standard regimens of antiepileptic drugs.

Ten (58.8%) patients received both adjuvant chemotherapy and radiation therapy, 2 patients (11.8%) received radiation therapy alone, and 5 patients (29.4%) did not undergo adjuvant therapy. The average length of follow-up was 6.7 months, and 4 (23.5%) patients passed away within the follow-up period. Seven GBM patients (53.8% of patients with GBMs) had documented recurrence of their tumor during the follow-up period.

#### 3.2.1. Case 1

**Figure 3:**
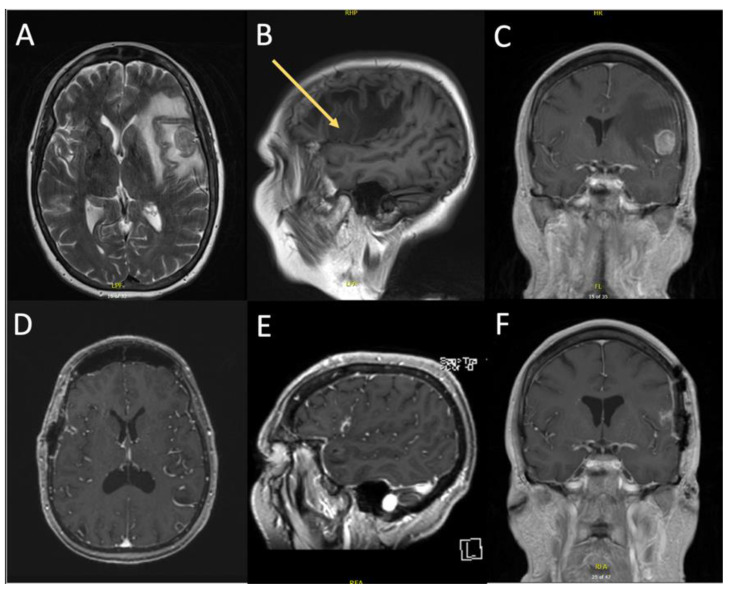
Preoperative MRI (top images) compared with 15-day postoperative MRI (bottom images). Image (**A**): Axial weighted T2 demonstrating extensive edema associated with lesion seen as a hyperintense signal with midline shift. Image (**B**): T1 MRI pre-Gd showing a hypointense, rounded, well-defined lesion within pars triangularis of the left inferior frontal gyrus (IFG). Vasogenic edema is visible as a hypointense signal. Image (**C**): T1-Weighted coronal view with contrast demostrating homogenous enhancement of the lesion and clear visualization of its margins. Ipsilateral collapsed lateral ventricle and midline shift secondary to surrounding hypointense edema is present. Image (**D**): Weighted T1 post-Gd with complete resection of the lesion. There is a small pneumocranium and a hyperintense signal beneath the craniotomy representing a small subarachnoid hemorrhage. Image (**E**): T1 weighted MRI showing a small hyperintense signal in the left IFG where the metastasis was previously located. Peritumoral edema is not present compared to before surgery. Image (**F**): Coronal weighted T1 post-Gd giving another view of the complete resection of the lesion with craniotomy, small hyperintense subarachnoid hemorrhage, and small pneumocranium. The previously collapsed left lateral ventricle is nearly symmetrical due to reduced edema.

We present a 55-year-old female with a previous history of stage IIIa (cT1b, cN2, cM0) non-small cell lung cancer (NSCLC), who experienced expressive aphasia, transient loss of coordination in the right upper extremity, and headache four days prior to surgery. Preoperative MRI revealed a 2.5 × 1.7 cm^2^ mass in the inferior gyrus of the left frontal lobe that was suspected to be a metastasis (Figure 3A–C). Surrounding edema and effacement of the left lateral ventricle were associated with the lesion. The patient underwent a left sided craniotomy utilizing IOUS and TScal for the tumor resection. Post operative recovery was uneventful, and the patient was released from the hospital 4 days after surgery. At the follow up visit 15 days later, the patient experienced complete resolution of her aphasia without any new deficits. Imaging indicated complete resection of the tumor and reduction of edema (Figure 3D–F).

#### 3.2.2. Case 2

**Figure 4:**
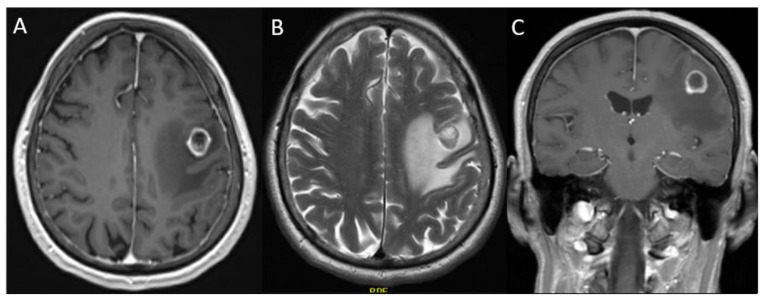
Preoperative MRIs. (**A**): Axial weighted T1 with contrast demonstrating a well circumscribed lesion in the left precentral gyrus with surrounding hypointense signal representing edema. (**B**): T2-Weitghed MRI with a hyperintense, ovoid, well-defined lesion within the left precentral gyrus. Vasogenic edema is visible in this image as a hyperintense signal around the mass. (**C**): Weighted coronal T1 with contrast demonstrating clear visualization of the lesions well-circumscribed margins.

**Figure 5:**
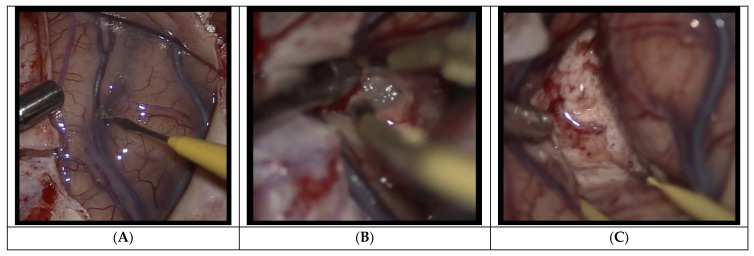
Demonstration of some steps of the trans-sulcal approach after opening the dura. (**A**): Opening of the precentral sulcus arachnoid. (**B**): Partial resection of the lesion after corticectomy in the floor of the sulcus. (**C**): Complete resection of the lesion with wide dissection of the sulcus clearly visible.

We present a 71-year-old female with a right upper lobe lung mass, who developed aphasia and progressive right-sided hemiparesis one month prior to the surgery. Following an ER admission, a pre-operative MRI revealed an enhancing IaT centered in the left pre-central gyrus measuring 1.4 × 1.4 × 1.0 cm^3^ with surrounding vasogenic edema (Figure 4). The patient underwent a wide TScal through the pre-central sulcus. The tumor was completely removed in an extra-capsular fashion (Figure 5). The patient was then discharged four days later. In the first follow-up visit 14 days later, the patient presented improvement of speech and hemiparesis. Pathology results indicated a KRAS-mutated lung adenocarcinoma.

## 4. Discussion

The microscopic TScal was first described by Yaşargil et al. in 1988, who hypothesized that the sulcal corridor could be safely utilized to avoid corticectomies in eloquent cortex [34]. Until recently, most researchers have focused on non-eloquent regions for this procedure and have noted few, if any, new deficits following surgery [45,46,47]. Mikuni and Hashimoto noted no new neurologic deficits while traversing anatomically eloquent regions in 2006 [46]. Farid et al. followed by reporting their experiences in 42 patients for non-eloquent and 26 eloquent lesions in 2019. Farid’s team reported a GTR rate of 52%, but improvement in preoperative deficits or development new of postoperative neurologic sequelae were not documented [48].

In this retrospective analysis, the authors report positive surgeon experience and results with the microsurgical TScal using wide dissection in eloquent regions. A GTR rate of 88% was achieved in this subject group, with wide dissection allowing for a clear visualization of malignant lesions. Despite operating in eloquent areas, 88.2% of patients experienced improvement in their preoperative deficits, 64.7% had full resolution of their neurological symptoms, and no patients experienced permanent new-onset neurological deficits. These improvements were seen despite the highly invasive nature of the lesions, eleven of which were GBM. Duffau previously described neuroplasticity associated with invasive glial tumors that may account for retained function in malignancy [49]. As neural tissue is increasingly seen as dynamic in structure and function, the adaptive changes of neuronal networks to malignancy may explain the neurological improvement seen in these patients. Furthermore, many lesions had significant reduction in large collections of vasogenic edema as noted in both illustrative cases, potentially enhancing functional recovery (Figure 4 and Figure 5). With these factors in mind, the data represents similar or greater GTR and postoperative improvements when compared to other TScal literature [48,50,51,52].

Follow-up mortality at 6 months was 23.7%, which was within mortality rates for GBM and other metastatic diseases [7,53,54]. Post-surgical complications include surgical wound infection in one patient (5.9%), hematoma in two patients (10.8%) and DVT in three (17.6%). These results are similar to other reports on TScal and for craniotomies in general, indicating no major change in risk [51,55,56,57,58]. Some authors on the TScal did not note DVT as a complication. This team’s experiences may be explained by the hypercoagulable state associated with malignancies, a well-documented concern in high grade gliomas [59,60,61,62]. As a result, the occurrence of DVTs was not unexpected in these participants.

There are some slight deviations in surgical outcomes between our study and others using the TScal. Some authors report slightly higher GTR rates, less surgical complications, and reduced mortality [46,51,58,63,64]. Influencing factors may include limited sample size of this study, lesion characteristics, and retraction methods. Research on the TScal often focuses on low grade and benign lesions such as meningiomas, colloid cysts, and low-grade gliomas. High grade gliomas and metastatic lesions comprised 70.6% and 22.6% of the cases our study, respectively, and all 4 patients who expired had Grade 4 glioblastomas. These high-grade lesions demonstrate a greater postsurgical morbidity and mortality compared to lower grade tumors [7,65]. Increased morbidity in this group may be reflected by the percentage of seizures experienced by participants. Postoperative seizures occurred in 35.3% of subjects, yet other case series recorded between 0% and 17.6% occurrence [63,66,67]. Current research indicates high grade gliomas may be a significant risk factor for postoperative seizures [68,69]. Out of the 6 subjects that experienced post-operative seizures, 5 (83.3% of patients who experienced seizures) were Grade 4 Glioblastoma patients. Seizure rates vary from 1.1% to 48%, if not even higher, for brain tumor resection overall [70,71,72]. As a result, the reported number of postoperative seizures in this study may reflect subjects in a high-risk demographic for seizure occurrence. Additionally, most of these authors focus on the use of tubular retractors during the TScal. Dynamic retraction was used, as wide dissection TScal requires extensive retraction and dissection of the cortical tissue to expose tumors located in the parenchyma. Local irritation associated with manipulation of cortex during has been proposed to explain to explain the increase in seizures following intracranial masses [73]. Considering substantial manipulation of brain tissue is characteristic of the wide-dissection TScal, local irritation of the cortex may have resulted in postoperative seizures in this patient group. Furthermore, ongoing research indicates tubular and traditional retraction have different postsurgical morbidity characteristics, including the occurrence of post-operative seizures [52,74,75,76]. However, it is difficult to conclude whether TScal predisposes patients to a higher incidence of postoperative seizures due to the sample size and lack of control group in this study. Further research directly comparing wide-dissection TScal to alternative retraction techniques, as well as comparisons of different primary and secondary tumor postsurgical outcomes in wide-dissection TScal, are needed to better contextualize these results.

Lesions located beneath eloquent cortex require careful navigation to avoid permanent neurological deficits. The advent of the awake craniotomy allowed for careful guidance during surgery to attempt to maximize function and surgical resection. However, not every patient is suitable for this technique. This procedure is often limited by patient communication abilities [77]. Language barriers, developmental deficits, patient confusion, pediatric patients, and intraoperative seizures can limit intraoperative communication and result in the awake craniotomy failing. Other considerations include patient history of anxiety, inability to sit still, learning difficulty, obesity, and uncomfortable surgical positioning [16,27,29,77,78]. The TScal offers a potential alternative to circumvent these limitations. As patients do not need to be awake and communicative during the procedure, those who are ineligible for awake craniotomies may be a candidate for the TScal. Furthermore, this group reports a percent GTR and post-surgical deficits that were comparable or lower than that observed in some awake craniotomies [79,80]. Considering these potential limitations associated with awake craniotomy, the data of this study support TScal as an alternative approach for eloquent cortex lesions.The TScal enables the use of other intraoperative tools that increase the surgeon’s ability to fully excise lesions. 5-ALA, a microscopic fluorescent guidance to increase residual tumor resection, has been investigated thoroughly in the literature and stage III clinical trials [81,82,83]. At this center, 5-ALA was employed in some cases to improve tumor margin resection while using the TScal (Figure 2). Another ancillary intraoperative tool employed during the TScal was IOUS, as demonstrated in Case 1. IOUS allows for compensation for “brain-shift”, or movement of the brain during craniotomies, that can hinder accurate neuro-navigation [84,85,86,87]. IOUS monitoring can be implemented during TScal to increase assurance of accurate dissection both before opening the dura, and while ensuring the ideal sulcus to use for the approach. Although the surgeons in this study did not utilize other modalities of intraoperative imaging, it is worth noting their utility and potential adaptation to the TScal approach. Kuhnt et al. described using diffusion tensor imaging (DTI) in combination with intraoperative magnetic resonance imaging (iMRI) to map and preserve white matter tracts with few post-operative deficits [88]. Utilizing intraoperative white matter tractography in combination with the theoretical framework of preserving functional cortex through the TScal may provide greater improvement in patient outcomes.

Caveats to TScal include the need to manipulate vasculature overlying the sulci and the severing of short association fibers. Latini and Ryttlefors have indicated that manipulation of vasculature to reach the sulcal floor risks local ischemic damage, potentially compromising cortex [89]. There was no indication of ischemic injury in this patient group even with wide dissection, but further investigation can clarify these risks. In using trans-sulcal corridor, severing short association fibers is necessary to visualize IaT (Figure 1). Although this theoretically could result functional losses, our results indicate no observable detriment caused to the patient by sacrificing these fibers, even in eloquent tissue. As mentioned previously, there were no measurable new permanent deficits and 88.2% of patients saw improvement in their preoperative symptoms. These findings corroborate observations from other authors that preserving the gyri and underlying projecting fibers may limit postoperative deficits, even when short association fibers are disrupted [90]. However, our postsurgical outcomes may have been influenced by the depth of the lesions in this population. There are no clearly defined parameters by which a lesion is considered to be located deep within the brain parenchyma. In our study, we classified deep lesions as being below the cortical-subcortical transition. Considering a median tumor depth of 6 mm in this sample of patients, tumors located further beneath the cortex may have worse surgical outcomes and presents a unique surgical challenge. This is corroborated by other studies using TScal with lesions located near the diencephalon resulted in decreased neurological symptom relief [50,51,52]. More shallow lesions within the parenchyma may enable optimal preservation of critical white matter tracts, potentially explaining the high number of patients who experienced relief from neurological symptoms. Further analysis with a greater sample size and greater variety of lesion depths within the white matter tracts may determine to what degree lesion location impacts the efficacy of TScal.

This study was limited by being a retrospective analysis of patient outcomes. As we did not have a control group to compare to, surgical outcomes had to be analyzed with respect to the literature. These limitations extend into patient selection, as the study included only a small cohort; namely subcortical lesions directly underlying an accessible sulcal corridor in eloquent areas where gyral corticectomy was impractical. Further investigation is needed to determine if this data correlates with the general population of patients who undergo trans-sulcal approach for subcortical malignant lesions.

## 5. Conclusions

The TScal is a viable alternative treatment for lesions in eloquent regions of the brain when patients are unable tolerate awake craniotomies. This approach conserves gyral cortex and the projecting fibers of the white matter, reducing preoperative neurological deficits and aiming to minimize postoperative morbidity. Wide dissection during TScal enables enhanced visualization of lesion margins when tubular retraction may limit field of view. Ancillary techniques including IOUS and 5-ALA can be employed to improve resection of lesions. This flexibility enables surgeons to tailor the treatment to the patient, as well as use many intraoperative tools that they are familiar with. Postoperative morbidity and mortality were comparable to using tubular retraction or awake craniotomy, without measurable deficits caused by disruption of sulcal vasculature, cortex, and short association fibers. Further analysis with deeper tumors and a larger sample size will determine how these postsurgical outcomes compare to the general population.

## Figures and Tables

**Table 1:** Patients with Diagnoses and Outcomes.

Patient Number	Age	Diagnosis	Comprehensive Preoperative Neurological Examination				Comprehensive Postoperative Neurological Examination				Length of Stay	Postoperative Complications
			C	M	S	Sp	C	M	S	Sp		
1	68	GBM	N	N	Y	Y	N	N	N	N	4 days	No
2	67	GBM	Y	N	N	Y	N	N	N	N	8 days	No
3	76	GBM	N	Y	Y	Y	N	N	N	N	5 days	Subdural hematoma, DVT
4	52	Lung Adenocarcinoma	Y	N	N	Y	N	N	N	N	2 days	DVT
5	75	GBM	N	Y	Y	Y	N	N	N	N	5 days	Seizures, DVT
6	46	GBM	N	N	N	N	N	N	N	N	2 days	No
7	57	GBM	N	N	N	Y	N	N	N	N	4 days	Seizures
8	56	GBM	N	Y	N	Y	N	Y	N	N	5 days	Seizures, Subdural hematoma
9	30	Diffuse Astrocytoma (WHO 2)	N	Y	N	N	N	Y	N	N	3 days	No
10	70	Anaplastic Astrocytoma (WHO 3)	Y	N	N	Y	Y	N	N	Y	3 days	No
11	64	GBM	Y	N	Y	Y	Y	N	Y	N	3 days	No
12	65	GBM	N	Y	N	Y	N	N	N	N	3 days	No
13	61	GBM	N	Y	N	Y	N	N	N	Y	3 days	Seizures
14	59	Lung Adenocarcinoma	N	N	N	Y	N	N	N	N	3 days	No
15	61	Small Cell Lung Carcinoma	N	Y	N	N	N	N	N	N	5 days	Seizures
16	73	Colon Adenocarcinoma	N	Y	N	N	N	N	N	N	4 days	Seizures
17	61	GBM	N	Y	Y	N	N	Y*	N	N	2 days	No

**Table 2:** Patient demographical data and tumor characteristics.

Patient Demographics.	N (Percentage)
**Total subjects Gender**	17
Males	10 (58.8%)
Females	7 (41.2%)
**Age**	
Range	21–76
Average	61.2
**Lesion Location**	
Left Precentral gyrus	3 (17.6%)
Right Precentral gyrus	4 (23.5%)
Left Postcentral gyrus	1 (5.9%)
Left Frontal Speech Area	2 (11.8%)
Left Posterior Speech Area	5 (29.4%)
Left Visual Cortex	1 (5.9%)
**Primary CNS Lesion**	
Grade 4 Glioblastoma	11 (64.7%)
Grade 3 Astrocytoma	1 (5.9%)
Grade 2 Diffuse Astrocytoma	1 (5.9%)
Total Primary CNS lesions	13 (76.5 %)
**Metastatic Lesion**	
Small Cell Lung Cancer	1 (5.9%)
Lung Adenocarcinoma	2 (11.8%)
Colorectal Adenocarcinoma	1 (5.9%)
Total Metastatic Lesions	4 (23.5%)

**Table 3:** Patient deficits before and following trans-sulcal tumor excision.

	Preoperative Deficits (N, Number; Percentage)	Postoperative Deficits (N, Number: Percentage)
Cognitive	4 (23.5%)	1 (5.8%)
Speech	12 (70.6%)	1 (5.8%)
Motor	9 (52.9%)	3 (17.6%)
Sensory	5 (29.4%)	1 (5.8%)

## Data Availability

The data presented in this study are available on request from the corresponding author. The study data collection form contains patient identifiers.
